# The influence of cultural friction on foreign divestment of multinational enterprises——the moderating role of formal institutional distance and political connections

**DOI:** 10.1371/journal.pone.0295443

**Published:** 2024-02-09

**Authors:** Zijing Xu, Ming Tian, Yang Zhang

**Affiliations:** School of Business, Hohai University, Nanjing, Jiangsu, China; Lingnan University, The University of HongKong, HONG KONG

## Abstract

Multinational enterprises frequently divest their foreign assets in the current economic environment. Existing research, based on friction theory, has mainly focused on the impacts of political and economic disparities on foreign divestment while neglecting the nuanced influence of cultural factors. To address this gap, this paper draws on the cultural friction perspective to capture the diverse cultural resistance faced by each enterprise and explore the relationship between cultural friction and foreign divestment. Data from Chinese publicly listed enterprises engaged in foreign investment are leveraged, and a dual-level analysis is conducted using Logit panel regression and Cox survival analysis to examine the relationship between cultural friction and foreign divestment from both the viewpoints of the parent company and the overseas subsidiary. Additionally, the paper examines the marginal factors that affect the relationship between them from an institutional perspective. The findings reveal that cultural friction has a positive influence on the propensity of multinational enterprises to divest from foreign markets. Interestingly, a "formal institutional distance paradox" is demonstrated in our study, and politically connected enterprises are found to be more vulnerable to foreign divestment due to the "curse of political affiliations".

## 1 Introduction

Withdraw from imperiled foreign assets is a common phenomenon within a company’s internationalization journey. For instance, Chinese enterprises, such as Sinopec, Chinalco, and CNPC, have lately withdrew from the United States market and the Wall Street Journal reported that Chinese firms have collectively divested $23.6 billion from overseas real estate sector. Foreign Divestment (FD), which refers to the reduction or complete divestiture of foreign subsidiaries [1], has emerged as a possible outcome of corporate operations. This can include sales, bankruptcy, spin-offs, and divestiture [2]. Although foreign direct investment (FDI) typically signifies a long-term commitment to a foreign operation [3,4], divestments are not uncommon. In fact, for every two overseas subsidiaries formed, one is likely to be disposed of [5].

As a natural process of multinational enterprises’ overseas operation, few scholars pay attention to foreign divestment, primarily because many MNEs are reluctant to disclose details about their foreign divestment, often considering it as business failure. This lack of transparency results in a scarcity of relevant data [6]. Nevertheless, it is essential to examine the causes of foreign divestment, as it significantly affects both the firm and the country at large. Foreign divestment has a twofold effect. On one hand, it is a common strategy for enterprises to restructure their overseas assets, potentially impacting the company’s entire global supply chain. On the other hand, it can hinder the home country from reaping several benefits associated with FDI, particularly in terms of technology transfer [7,8].

In this paper, we focus on examining the determinants of foreign divestment. For a long time, scholars have paid more attention to the predictive role of political signals and economic factors on enterprises and market behaviors [9,10]. However, cultural factors have started to emerge as influential determinants in recent years. The study conducted by An, Hou & Lin delves into the cultural aspect of mistrust within financial institutions, revealing its detrimental impact on financial development [11]. Meanwhile, Xu et al. explored the intricate relationship between inherited trust and informal finance, discovering that trust significantly increases businesses’ reliance on trade credit [12]. Additionally, research has unveiled the influence of gender bias culture on firm financing, indicating disparities between men and women’s access to trade credits in international markets and their propensity to seek informal financial support [13]. Moreover, An, Jiang & Xu explained the heterogeneous effects of normative culture on business risk-taking behavior based on social identity theory and peer effect theory [14]. These studies collectively emphasize the critical role of cultural factors in shaping various aspects of firm behavior. Notably, despite these advancements, there exists a relatively unexplored area, namely the impact of culture on multinational enterprises’ foreign divestment. However, our data reveals that 70% of divestments are connected to cultural factors, underscoring the practical implications of considering foreign divestment from a cultural perspective.

In the field of international business research, a single cultural dimension often falls short in comprehensively measuring the impact of cultural factors on corporate behavior. Consequently, the theory of cultural distance, which incorporates multiple cultural factors, has gained widespread recognition within the academic community. This concept, initially proposed by Hofstede, pertains to the extent of variations in shared norms and values among countries, encompassing six dimensions: power distance, individualism, masculinity, uncertainty avoidance, long-term orientation, and indulgence [15]. Currently, cultural distance serves as a valuable tool for investigating various aspects, including the location choice for international investments, the selection of entry modes, and the performance of overseas subsidiaries [16]. Nonetheless, a wealth of empirical data underscores the existence of the cultural distance paradox [16,17]. For example, some scholars contend that cultural distance plays a pivotal role in the dissolution of international joint ventures, while others argue that it has a positive impact on the formation and performance of international joint ventures, thereby leaving managers in a dilemma [18,19]. In fact, the distance theory assumes that firms with diverse characteristics encounter similar disparities within the same host country environment, which is inherently biased. For example, Bao & Huang pointed out that the same external shock could lead to entirely different outcomes for different firms [20]. Therefore, how to transform macro factors into micro data in order to discover the heterogeneous differences faced by various enterprises in the same host country becomes the key to such research.

In recent years, many scholars have explored ways to transform macro data into more detailed micro-level data. For instance, Yu & Huang have introduced a novel approach that combines enterprise data with market data, providing a fresh perspective for our study [21]. In line with the above practices, scholars have put forth the concept of cultural friction by blending national cultural distance with enterprise micro-data. As described by Luo & Shenkar, cultural friction (CF) is a consequence of cultural differences and represents the cultural resistance that arises during interactions between multinational enterprises. As an advancement of the distance theory, cultural friction effectively merges national macro data with corporate micro data, enabling a more comprehensive understanding of the varied challenges faced by enterprises operating within the same international market. As a reverse process of outward expansion, foreign divestment is inevitably influenced by cultural contexts, and compared with the stable cultural distance, cultural friction considering the interaction between enterprises and the host country is more suitable for foreign divestment research. Therefore, this paper adopts a friction-based perspective to investigate how cultural resistance at the individual level impacts the foreign divestment decisions of multinational enterprises.

Additionally, we delve into the boundary factors that influence the connection between cultural friction and foreign divestment. Cultural friction, which serves as a tangible expression of national informal institutions at the firm level, is inherently constrained by both formal and informal institutions. Firstly, cultural friction arises from cultural distance and to some extent is the product of state informal institutions, making it susceptible to the influence of formal institutions. How to stimulate the positive interaction of formal institutions on the process of culture shaping is worthy of scholars’ attention, but previous studies have focused more on their respective mechanisms [22], neglecting their complementary relationship. Consequently, this paper introduces the concept of formal institutional distance to investigate its impact on the relationship between cultural friction and foreign divestment. Secondly, from the perspective of the informal system, political connections, a significant manifestation of informal ties, refer to the voluntary relationships that enterprises establish with domestic political entities [23]. Such connections are widespread in our country’s commercial culture. Presently, many enterprises employ political resources to mitigate disadvantages arising from informal factors like cultural differences [24]. However, the evolving dynamics between politics and business have unveiled the downsides of political connections. Specifically, excessive government funding and political demands placed on enterprises may lead to overconfidence in blind investments, exacerbate the legitimacy challenges faced by transnational enterprises, and subsequently ensnare these enterprises in operational difficulties [25]. In order to explore the impact of informal institutional relationships on cultural friction and foreign divestment, this paper incorporates political connections to illustrate the constraints imposed by informal institutions.

Finally, considering that foreign divestment is a strategic decision made by the parent company based on its own development requirements as well as the individual characteristics of overseas subsidiaries, it will be influenced by both parent company factors and subsidiary factors. Therefore, we take a novel approach by analyzing the relationship between cultural friction and foreign divestment from both the parent and subsidiary levels. Furthermore, drawing inspiration from a study conducted by Yu & Huang, we acknowledge that the same antecedent variable may produce different effects on the same dependent variable over different time spans [26]. To address this, we employ panel data at the parent company level and cross-sectional data at the subsidiary level, allowing us to explore the long-term and short-term impacts of cultural friction. This approach represents a bold and valuable endeavor in this study, significantly advancing our understanding of foreign divestment research.

The structure of this paper is organized as follows: In Part Two, we conduct a theoretical analysis, reviewing relevant literature and presenting our research hypotheses. Part Three provides an overview of our research design, including data sources, variable measurement, and model construction. In Part Four, we delve into empirical analysis at the parent company level. Here, we thoroughly investigate the impact of overall corporate cultural friction on divestment probability. This section includes descriptive statistics, regression analysis, and robustness checks. Part Five shifts the focus to subsidiary-level analysis. We explore how the unique cultural friction experienced by each subsidiary influences its survival probability. This examination involves descriptive statistics, non-parametric analysis, and Cox regression. Finally, in Part Six, we sum up our research findings and engage in a discussion of their implications and significance.

## 2 Literature review and research hypothesis

### 2.1 The upgrade from "cultural distance" to "cultural friction"

In the field of international business research, cultural distance, as an objective measurement of cultural differences, has found widespread use [27]. Over the past few decades, it has undergone a transformation from a one-dimensional concept to a multidimensional construct, evolving in step with the development of integrated measurement methods for cultural dimensions. This evolution has contributed to a more comprehensive understanding of cultural distance. Nonetheless, recent years have witnessed a growing chorus of scholars expressing concerns about the conceptual shortcomings and methodological limitations of distance theory, which has led to the emergence of the theory of friction [28]. Differing from distance theory solely emphasizes national disparities, friction theory shifts research focus from abstract differences to the practical realm of entity interactions. By adeptly melding national and firm-level differences, it establishes a more robust framework for capturing the behavioral characteristics of multinational enterprises. This transition can effectively address the shortcomings of distance theory [29].

In the realm of quantitative research, the task of enhancing distance theory and devising measurement methodologies to gauge the levels of friction among diverse entities is undoubtedly a substantial challenge. Using the distance theory as a foundation, Professor Luo established a quantitative research paradigm for measuring cultural friction in 2011.This paradigm included physical friction components like velocity, stage, and contact surface, which represent the degree, speed and stage of internationalization of enterprises. By incorporating these physical friction elements, the paradigm demonstrates its ability to capture the diverse cultural differences faced by individual firms, effectively surmounting the limitations inherent in distance theory. As a result, this research is poised to employ this innovative measurement paradigm in relevant investigations, marking the way for a more comprehensive analysis of cultural friction in the field of international business research.

### 2.2 The relationship between cultural friction and foreign divestment

Currently, scholars frequently approach the study of factors influencing foreign divestment from various perspectives, including transaction cost theory, resource-based view, institutional theory, and real option theory. This has led to the preliminary formation of a theoretical framework categorized by company and country characteristics [30]. Cultural distance, a widely used theory to capture cultural disparities between home and host countries, has gained significant traction in the field of international business. However, empirical research on cultural distance has yielded contradictory results. While some scholars argue that countries with greater cultural distance face higher divestment risks in comparison to countries with closer cultural affinity [31], others have arrived at contrasting conclusions [32,33]. Foreign divestment, as a strategic decision made by parent companies based on host environments and company-specific attributes, emerges from the combined influence of internal factors within firms and external environmental conditions. This makes us wonder if the concept of cultural distance, which predominantly considers national disparities, can accurately convey the underlying motivations behind divestment. Compared to distance theory, friction theory provides a unique perspective on the interactions between nations and firms. It effectively captures the heterogeneous differences in the overseas investment, rendering it an excellent research viewpoint for this study.

Scholars have long held that differences between the home and host countries’ political, economic, cultural, and other external environments are the primary sources of the liability of foreignness (LOF). This theory is frequently employed to elucidate the adverse effects of these disparities on the overseas operations of multinational corporations [34]. The liability of foreignness theory, initially introduced by Zaheer, pertains to the competitive disadvantages faced by multinational firms due to their foreign status in the host country market. These disadvantages primarily result from the additional operating costs incurred by multinational operations. LOF can be broadly categorized into three components: unfamiliarity costs, relational costs, and discrimination costs [35]. Unfamiliarity costs primarily stem from the incomplete understanding of the host country market norms, cultural customs, and other information provided by multinational enterprises. This is exacerbated by the misjudgment of these entities by host country stakeholders, which, in turn, results from information asymmetry between transaction parties [36]. On the other hand, relational costs and discrimination costs predominantly arise from the legitimacy deficit faced by multinational enterprises in the host country market. This encompasses various dimensions, including regulatory, normative, and cognitive legitimacy deficits [37,38].

Cultural friction, as a specific manifestation of national cultural differences at the level of individual enterprises, similarly faces a dual liability of foreignness involving information asymmetry and legitimacy deficit, thereby increasing the risk of foreign divestment. Firstly, in terms of information asymmetry, cultural friction exacerbates the informational disadvantage faced by foreign enterprises in comparison to local stakeholders. On one hand, cultural friction increases the difficulty for multinational enterprises to acquire and comprehend market information in the host country, necessitating more information gathering and knowledge-building efforts [39], consequently raising the unfamiliarity costs for these firms. On the other hand, some host countries frequently rely on impressions to judge the identity and strategic goals of foreign investors [40], which makes it more difficult for multinational corporations to establish relationships with local stakeholders, therefore increasing the costs of discrimination and relationship maintenance [41]. Secondly, from the standpoint of legitimacy deficit, cultural differences in values and ideologies can result in cognitive legitimacy deficits for multinational enterprises, deepening the mistrust of local stakeholders [40]. This mistrust has a twofold impact: first, it amplifies the uncertainty and volatility of cross-border investments, compelling firms to address the issue of insufficient trust through contract arrangements and similar means, affecting operational efficiency. Second, this mistrust forces multinational enterprises to discriminatory treatment and hinders their resource acquisition processes, which, in turn, forces them to incur additional costs in conveying positive signals to the external environment to secure cognitive legitimacy within the host country market [42]. In conclusion, whether viewed through the lens of information asymmetry or cognitive legitimacy deficit, the individual cultural differences represented by cultural friction augment the survival costs of multinational enterprises, resulting in foreignness disadvantage [43], consequently triggering foreign divestment. Therefore, this paper proposes the following hypotheses:

H1: Cultural friction positively influences the foreign divestment of multinational enterprises.

### 2.3 The moderating role of formal and informal institutions

Foreign divestment has a multi-national context and is closely related to institutional factors. Institutions, often described as "rules", can be categorized into formal and informal arrangements within the framework of New Institutional Economics. Formal institutions encompass political, legal, and regulatory structures, while informal institutions relate to unwritten norms developed through social interactions [44]. This perspective underscores that organizations are subject to the dual constraints of formal and informal institutions, which offers a novel theoretical lens to examine the boundary factors influencing the relationship between cultural friction and foreign divestment. In current academia, the concept of formal institutional distance is widely used to represent the disparities in formal institutions that multinational enterprises encounter [45], and informal institutional arrangements such as political connections are considered significant strategies for firms to overcome cultural barriers [24]. Consequently, this study takes an institutional viewpoint, employing the concepts of formal institutional distance and political connections to investigate the impacts of both formal and informal institutional arrangements on the relationship between cultural friction and foreign divestment.

#### 2.3.1 The moderating effect of formal institutional distance

Following the conventional institutional logic, formal institutional distance will negatively impact a firm’s international expansion [43]. This suggests that businesses tend to operate in countries with smaller differences in formal institutions. Historically, scholars have employed transaction cost theory to explain this phenomenon, asserting that institutional disparities increase a firm’s overseas operational costs and reduce the success rate of foreign investments [46]. However, with the emergence of the new institutional theory, the "reverse logic" of institutional distance has gradually gained recognition in academia. From the perspective of the real options theory within the new institutional framework, differing institutional contexts are viewed as fertile ground for organizations to effect changes. This, in turn, allows enterprises to explore new market opportunities by capitalizing on institutional voids [47,48]. For example, Dunning & Lundan put forth the argument that the institutional conditions of certain locations may offer better opportunities for exploring specific types of ownership advantages. Institutional arbitrage, they posited, could become the primary driving force behind globalization [49].

Currently, many scholars have empirically demonstrated the moderating role of formal institutional distance in the relationship between cultural differences and foreign investment [50,51]. However, research on how formal institutional distance affects the relationship between cultural friction and foreign divestment remains limited. To fill this gap, formal institutional distance is included in this study as a moderator variable. Firstly, adhering to traditional institutional logic, formal institutional distance amplifies the cost disadvantage resulting from cultural friction. Given the irreversibility of investments, this study suggests that both sunk costs and future reentry fees would exert a "hysteresis" effect on firms’ divestment decisions [52,53]. Secondly, following the logic of the new institutional theory, formal institutional distance increases the uncertainty in a firm’s operations. This unfavorable environment creates a "zone of inaction" where firms are inclined to maintain decision inertia amidst adverse circumstances [54]. In these situations, formal institutional distance will negatively impact the positive effect of cultural friction on foreign divestment. Thus, this paper proposes the following hypotheses:

H2: Formal institutional distance diminishes the positive impact of cultural friction on foreign divestment.

#### 2.3.2 The moderating effect of political connections

Political connections, as an informal mechanism, are prevalent in the context of Chinese commercial culture. Over time, this informal institution has become recognized as a crucial means for firms to secure vital resources, with many companies voluntarily appointing political figures to key positions in the hopes of building a protective shield through political means. In such instances, political connections are seen as an alternative mechanism for safeguarding corporate interests and enhancing market value [55]. However, when companies excessively prioritize the value of political-business relationships, the negative implications of political connections gradually come to the forefront. Firstly, from a cost perspective, companies seeking political resources need to bear certain rent-seeking costs, which to some extent deplete their available capital. With the increasing intensity of China’s anti-corruption efforts and the rapid development of the market economy, establishing and maintaining political connections have become increasingly challenging, resulting in gradually escalating costs [56]. Taking Chinese companies as an example, scholars point out that companies with political connections are more susceptible to the impact of political scandals and corruption factors compared to ordinary companies, thus exerting a negative influence on their stock prices [57]. Secondly, from a performance perspective, companies with political connections are more likely to cater to the social demands of the government and politicians, which can lead to a misallocation of resources [25]. In this context, political connections become a political burden, subjecting companies to the invisible hand of the government and hindering their normal investment and financing activities, ultimately resulting in the "political resource curse effect" [58,59].

Scholars have predominantly explored the topic of political connections in foreign markets with a focus on overseas mergers and acquisitions. Some researchers view political connections as a driving factor behind firms engaging in foreign investments, contending that these connections can mitigate the negative impact of informal institutional distance on the performance of overseas mergers and acquisitions [60]. Conversely, other scholars examining the relationship between merger experience and cross-border merger performance find that political connections exert a reverse effect. In this context, the "political label" exacerbates the discrimination costs for multinational enterprises, resulting in a resource curse [61]. Moreover, a literature search reveals a consistent association between political connections and "over-investment". Essentially, the higher the degree of political correlation of an enterprise, the greater the level of over-investment [59,62]. This phenomenon is attributed to government policy biases and financial assistance that foster unwarranted confidence in blind expansion, leading to issues such as low investment efficiency and insufficient caution. As foreign divestment is a potential outcome of overseas investments, it demands careful consideration. Therefore, this study incorporates political connections into the analysis of foreign divestment to validate their influence on the relationship between cultural friction and foreign divestment. Thus, this paper proposes the following hypotheses:

H3: Political connections intensify the positive impact of cultural friction on foreign divestment.

**Fig 1:**
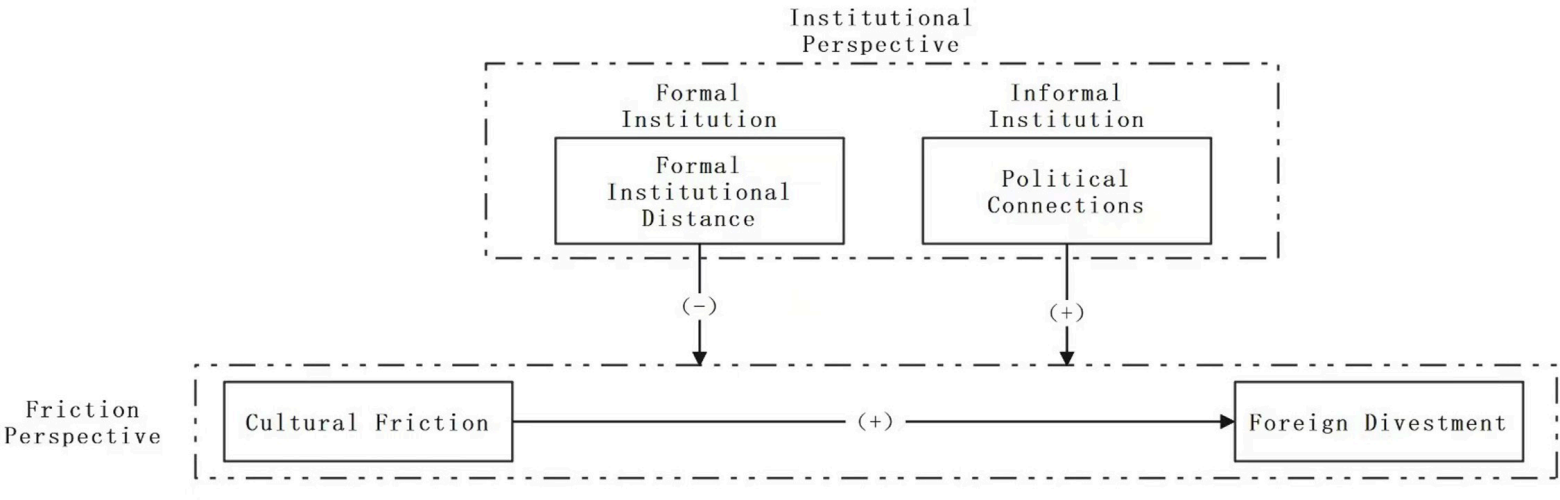
Research framework.

Building upon the analysis presented above, this paper proposes the following research framework (Fig 1).

## 3 Data and methodology

### 3.1 Data and sample

The empirical data for this study are based on the A-share listed Chinese MNEs’ FDI information spanning from 2012 to 2020. We meticulously collected this information from the CSMAR database and carried out a systematic investigation, including the scrutiny of annual reports, official statements, and direct contact with the investing businesses, to identify divestment of foreign units. This comprehensive process yielded a sample comprising 265 parent companies and a list of 351 overseas subsidiary exits of Chinese MNEs. Details of the data can be found in S1 Dataset. Chinese MNEs serve as an excellent research context for two significant reasons. First, Chinese enterprises have established an economy with a strong focus on exports and a diverse range of investment target nations, providing a substantial pool of samples for our research. Second, Chinese listed companies offer relatively complete data, as most data is mandated to be released, ensuring the availability of our research data. To mitigate the limitations of single-country data, we have taken FDIs with varying degrees of firm interaction into account, such as the number of subsidiaries and the pace and sequence of internationalization. This serves as the basis for the measurement of cultural friction. All the data are arranged as a panel dataset, and the 2,385 data from 2012 to 2020 of 265 Chinese overseas enterprises make up our sample.

### 3.2 Variable measurement

#### 3.2.1 Dependent variable

The dependent variable in this study is foreign divestment, which is used to evaluate instances of multinational enterprises’ parent companies retracting their overseas subsidiaries. Drawing inspiration from the methodology outlined by Nguyen et al., this research employs binary indicators to quantify whether overseas subsidiaries withdraw from foreign markets. In this binary system, 1 is assigned to cases of divestment events, while 0 corresponds to situations where no divestment event occurred. The relevant data for this analysis is sourced from the CSMAR database, which provides fundamental information on listed companies. The finalized dataset comprises a total of 265 Chinese listed companies and encompasses 351 incidents of foreign divestment that occurred within 55 host countries.

#### 3.2.2 Independent variable

In this study, the independent variable is cultural friction, which is utilized to evaluate the overseas market resistance faced by multinational enterprises. Following the quantitative research approach outlined by Luo & Shenkar, this paper integrates the elements of physical friction, encompassing factors such as velocity, stages, and contact surface, to formulate the following equation:

$$CF=\frac{N\cdot e^{V(1-G)}\cdot CD}{10}$$ (1)

In which, *CF* is the levels of cultural friction faced by individual enterprises. *N* is the cumulative sum of active overseas investments held by the parent company in the corresponding year. *V* is the increment in the number of overseas investments by the parent company in the corresponding year. *G* is the relative pace of internationalization for multinational enterprises, assigning 0 to the initial year of internationalization and adopting the ratio of yearly incremental investments to the cumulative investment for subsequent years. *CD* is the cultural distance between the home country and the host country.

As an evolution of distance theory, the measurement of cultural friction is closely intertwined with the foundations of distance theory. The complete equation consists of two components, which are used to assess disparities at the national and corporate levels, respectively. In the context of national distinctions, Hofstede’s cultural dimensions are employed for computation, encompassing six dimensions: power distance, individualism, masculinity, uncertainty avoidance, long-term orientation, and indulgence. These dimensions are amalgamated through the application of the standard Euclidean distance [63]. The precise formula is delineated below:

$$CD_{ij}=\sqrt{\sum_{k=1}^{n}\frac{{\left(I_{ki}-I_{kj}\right)}^{2}}{V_{k}}}$$ (2)

In which, *CD*_*ij*_ is the cultural distance between host country *i* and parent country *j*. *I*_*ki*_ is the score of host country *i* on the *k*th cultural dimension, while *I*_*kj*_ is the score of parent country *j* on the same *k*th cultural dimension. *V*_*k*_ is the variance of cultural dimension scores across all host countries for the *k*th dimension. *n* is the number of cultural dimensions.

This study introduces an innovative approach by integrating the cultural friction resistance faced by each subsidiary to the parent company level. It recognizes that the parent company has the final authority over the removal of its overseas subsidiaries, and the decision whether to divest is contingent on the parent company’s overall operational status. This integration aims to measure whether the cumulative cultural friction resistance encountered by the parent company influences its decisions on foreign divestment. Furthermore, this research focuses on the longitudinal cultural friction data of the parent company, with the intention of capturing the dynamic process in which cultural friction transitions from quantitative to qualitative impacts on foreign divestment decisions. Given that multinational corporations frequently have subsidiaries spread across several nations and must contend with the cultural interweaving with multiple host countries, integrating cultural friction at the parent company level can, to some extent, avoid the issue of little variability of annual cultural friction, which is of more practical significance.

#### 3.2.3 Moderator variables

1. In this study, we utilize the concept of formal institutional distance (*Inst*) to delineate the differences in formal institutional frameworks between the home country and host country. This characterization serves to quantify the external environmental distinctions faced by different enterprises. To assess formal institutional distance, we employ six sub-indicators from the global governance indices in accordance with accepted standards [64]. These sub-indicators encompass voice and accountability, political stability and no-Violence, government effectiveness, regulatory quality, rule of law, and control of corruption. The data is sourced from the WGI database, and the specific measurement formula is provided as follows:
$$Inst=\sqrt{\sum_{k=1}^{N}{\left(I_{ki}-I_{kj}\right)}^{2}}$$ (3)
  In which, *Inst* is the formal institutional distance between two nations. *I*_*ki*_ is the score of country *i* on the *k*th dimension, *I*_*kj*_ is the score of country *j* on the same *k*th dimension. *N* is the number of dimensions in the institutional environment.
2. The term "political connections" (*PC*) characterizes the informal institutional arrangements that enterprises autonomously establish to mitigate the adverse impacts of external cultural resistance. The measurement approach adheres to the methodology developed by Francis et al., which stipulates that an enterprise is considered to possess political connections if any of its board members have held or currently hold government positions, such as being a National People’s Congress representative, Chinese People’s Political Consultative Conference member, or local government official [65]. Subsequently, we employ a binary variable, assigning a value of 1 if the enterprise has political connections, 0 otherwise.

#### 3.2.4 Control variables

1. Firm Size (*Size*): Aberrations in firm size are considered pivotal factors influencing decisions regarding foreign divestment [66]. Previous research often measures firm size from perspectives such as total assets, total sales, and the number of employees. Total sales, as a crucial indicator of business performance, is a key factor affecting an enterprise’s entry into or exit from the market [54], which is more impartial than other indicators [67]. Therefore, this study employs the natural logarithm of total sales as the proxy to assess firm size.
2. Firm Age (*Age*): As an irreversible characteristic of a company, firm age is frequently used as a proxy for operational efficiency and competitiveness, which is vital in the internationalization of an enterprise [68,69]. Firstly, older businesses typically possess superior capacities for acquiring knowledge, managing risks, and mitigating negative effects. Secondly, these businesses often exhibit stronger investment vigor and have an advantage in acquiring political resources and establishing legitimacy abroad [70,71]. Consequently, firm age is included in the model as a key control variable.
3. Internationalization Experience (*Exp*): The significance of accumulating internationalization experience is widely recognized in diminishing uncertainties and enhancing a company’s ability to navigate external environmental fluctuations, which in turn can affect the survival rate of foreign subsidiaries [3]. Therefore, this study considers it as a crucial factor to control for when assessing the foreign divestment of multinational enterprises. Drawing on the approach of Chen & Hsu, the total count of overseas subsidiaries owned by the target enterprise in the years preceding the observation year is used to measure the accumulation of internationalization experience for the focal enterprise [72].
4. Firm Ownership (*Own*): Scholars have pointed out that the ownership of the firm is a critical variable influencing a company’s overseas behaviors. For instance, the distinct identity of state-owned multinational enterprises significantly impacts their choices in overseas market entry modes and subsequent operational performance [73,74]. Therefore, this study introduces the firm ownership variable into the model, employing a binary indicator for measurement. According to the previous research, state-owned multinational enterprises were coded as 1, and 0 otherwise [75].
5. Industry Property (*Ind*): After reviewing nearly three decades of research on foreign divestment, Arte & Larimo emphasized the importance of industry-level elements like technological innovation, industrial clusters, and structural relatedness for the survival of overseas subsidiaries [76]. Additionally, industry property plays a significant role in determining the expenses of foreign investment and the rate of internationalization [77]. Therefore, we classify target firms according to the Global Industry Classification Standard (GICS) and include it as a crucial control variable in the model. A complete summary of these indicators is presented in Table 1.

**Table 1:** Variable description and data source.

Variable type	Variable name	Variable symbol	Variable declaration	Data source
**Dependent variable**	Foreign divestment	*FD*	Dummy variable, 1 is assigned to the occurrence of *FD*, 0 otherwise	Basic information database of CSMAR listed companies
**Independent variable**	Cultural friction	*CF*	Calculated by formula (1)	List of overseas investments of CSMAR listed companies; Hofstede official website
**Moderator variable**	Formal institutional distance	*Inst*	Calculated by formula (3)	WGI database
	Political connections	*PC*	Dummy variable, 1 is assigned to political connections, 0 otherwise	CSMAR database
**Control variable**	Firm size	*Size*	Select the total sales confirmed by the operating year and do logarithmic processing	
	Firm age	*Age*	The natural logarithm of the numerical difference between the observation year and the established age	
	Internationalization experience	*Exp*	Total number of overseas subsidiaries in each year prior to the observation year	CSMAR database
	Firm ownership	*Own*	Dummy variable, 1 is assigned to state-owned enterprise, 0 otherwise	
	Industry property	*Ind*	According to GICS	

*Note*: This table presents summarized information on the variables. Columns 1 to 5 report variable type, variable name, variable symbol, variable declaration, and the data source, respectively.

### 3.3 Model selection

Since the dependent variable in this study is binary, and the binary logit regression model is commonly employed in the field of divestment, we have chosen it to estimate the odds of a firm being divested (Divestment) relative to not being divested [6]. Binary logit regression, as opposed to conventional linear regression, can improve predictive accuracy and reveal the probability of outcome occurrence, providing more insightful interpretations of the model’s coefficients. After conducting the Hausman test, we found that the sample data at the parent company level is best suited for the panel random effects Logit model. Model 1 comprises the explanatory variable "cultural friction" and the explained variable "foreign divestment." Model 2 expands on Model 1 by incorporating all control variables. Model 3 builds upon Model 2 by introducing an interaction term between formal institutional distance and cultural friction to assess its moderating effect on the main effect. Models 4 and 5, through grouped regression, verify the moderating role of the political connections, which represents informal institutional network relationships. The specific formulation of the models is as follows:

$$FD=\alpha_{1}+\beta_{1}\cdot CF+\delta_{1}$$ (4)

$$FD=\alpha_{2}+\beta_{2}\cdot CF+\gamma_{1}\cdot CV+\delta_{2}$$ (5)

$$FD=\alpha_{3}+\beta_{3}\cdot CF+\beta_{4}\cdot Inst+\beta_{5}\cdot CF\cdot Inst+\gamma_{2}\cdot CV+\delta_{3}$$ (6)

$$FD=\alpha_{4}+\beta_{6}\cdot CF+\beta_{7}\cdot PC+\gamma_{3}\cdot CV+\delta_{4}$$ (7)

$$FD=\alpha_{5}+\beta_{8}\cdot CF+\beta_{9}\cdot PC+\gamma_{4}\cdot CV+\delta_{5}$$ (8)

In which, *FD* is whether the enterprise engages in divestment. *CF* is the level of cultural friction faced by the enterprise. *Inst* is for the moderator variable formal institutional distance. *CF·Inst* is the interaction term between cultural friction and formal institutional distance. *PC* is the moderator variable political connections. *CV* is the control variables. *δ* is the random error term.

## 4. Result analysis

### 4.1 Descriptive statistics

To ensure the robustness of the analysis, this study employs a 1% trimming method on the variables to mitigate the potential influence of outliers on the analytical results. Subsequently, the data undergoes a descriptive analysis in Stata, encompassing foreign divestment, cultural friction, political connections, formal institutional distance, and all controlled variables, the details of which are presented in Table 2. The results of the descriptive statistics reveal that the mean divestment of the sampled enterprises is 0.147, with a standard deviation of 0.354. This slight deviation from the overall mean indicates relative consistency within the dataset. Notably, cultural friction demonstrates substantial variability, ranging from a minimum of 3.120 to a maximum of 16.902, signifying diverse levels of friction encountered by the sampled enterprises. Meanwhile, internationalization experience spans from 0 to a maximum of 403, with a standard deviation of 28.471, highlighting the uneven and significant differences in the degree of internationalization experience accumulation among the sample enterprises. Correlation analysis and collinearity test of the data are shown in S1 Table.

**Table 2:** Descriptive statistics of variables.

Variable name	Variable symbol	Sample size	Average	Standard deviation	Minimum	Maximum
Foreign divestment	*FD*	2385	0.147	0.354	0	1
Cultural friction	*CF*	2385	2.418	3.120	0	16.902
Firm size	*Size*	2385	22.166	1.633	17.060	26.846
Firm age	*Age*	2385	3.087	0.252	2.485	3.738
International experience	*Exp*	2385	14.701	28.471	0	403
Firm Ownership	*Own*	2385	0.200	0.400	0	1
Industry Property	*Ind*	2385	4.706	3.741	1	19
Formal institutional distance	*Inst*	2385	3.031	1.766	0	5.992
Political connections	*PC*	2385	0.325	0.469	0	1

*Note*: This table presents descriptive statistics for all variables, with columns 1 to 7 representing variable name, variable symbol, sample size, average value, standard deviation, and Max-min value, respectively.

### 4.2 Benchmark regression and moderating effect test

Binary Logit regression, unlike the conventional linear regression, requires the dependent variable to be a dichotomous binary variable. This prerequisite aligns with the setup of the present study, wherein the dependent variable pertains to divestment/non-divestment. The results of the regression analysis are presented in Table 3. In Model 1, which includes only the independent variable of cultural friction and the dependent variable of foreign divestment, the outcomes reveal a significant positive correlation between cultural friction and foreign divestment among multinational enterprises. The regression coefficient is denoted as *β*_*CF*_ = 0.100 (P < 0.001). Additionally, based on the odds ratio (OR) values, it is discerned that for every 1% increase in cultural friction, the likelihood of foreign divestment by multinational enterprises increases by 10% (P < 0.001). This finding implies that as the level of cultural friction faced by a company intensifies, the probability of foreign divestment also rises, thereby substantiating hypothesis H1.

**Table 3:** Benchmark regression and moderating effect test results.

Model/Variable	Benchmark regression		Regulatory effect test		
	(1)	(2)	(3)	(4)	(5)
	Full sample	Full sample	Full sample	*PC* = 0	*PC* = 1
**Explanatory variable**					
***CF***	0.100^***^ (0.000)	0.050^*^ (0.026)	0.340^***^ (0.000)	0.018 (0.532)	0.094^*^ (0.018)
**Moderator variable**					
***Inst***			0.288^***^ (0.000)		
***Inst·CF***			-0.085^***^ (0.000)		
**Control variable**					
***Size***		0.111^**^ (0.009)	0.088^*^ (0.043)	0.108^*^ (0.036)	0.081 (0.319)
***Age***		-0.925^***^ (0.000)	-0.899^***^ (0.000)	-1.069^**^ (0.001)	-0.542 (0.725)
***Exp***		0.004^+^ (0.051)	0.005^*^ (0.026)	0.009^*^ (0.017)	0.001 (0.725)
***Own***		-0.047 (0.778)	0.042 (0.805)	-0.047 (0.815)	-0.176 (0.597)
***Ind***		-0.009 (0.580)	-0.003 (0.843)	-0.012 (0.545)	0.001 (0.971)
**Constant**	-2.034^***^ (0.000)	-1.497 (0.179)	-2.015^+^ (0.075)	-0.897 (0.511)	-2.232 (0.265)
***LR***	38.91	54.45	94.45	39.89	19.03
***Prob >chi2***	0.000	0.000	0.000	0.000	0.004
***R*** ^***2***^	0.020	0.028	0.048	0.030	0.031

*Note*: This table presents the test results of benchmark regression and moderating effect in logit regression model. The coefficients in column 2 are obtained from the regression of cultural friction and foreign divestment. Similarly, Column 3 reports the regression coefficients of the variables after including all the control variables, column 4 reports the regression coefficients after the inclusion of formal institutional distance, and columns 5–6 report the regression results of the variables in groups without and with political connections, respectively. The p-values associated with these coefficients are reported in parentheses.

^+^p<0.1

^*^p<0.05

^**^p<0.01

^***^p<0.001.

Model 2, which includes all control variables, expands on the framework of Model 1. The results reveal a significant positive correlation between cultural friction and foreign divestment of multinational enterprises (*β*_*CF*_ = 0.050, P < 0.05). Additionally, the odds ratio (OR) value is 1.051, which is greater than 1, signifying that cultural friction amplifies the likelihood of foreign divestment, further affirming Hypothesis H1. Among all controlled factors, the variable of firm age (*Age*) exerts the most substantial impact on foreign divestment, with a regression coefficient of -0.925 (P < 0.001). This indicates that the longer a company’s operational history, the lower the probability of foreign divestment, a deduction consistent with the preceding analysis. However, it is imperative to note that the accumulation of internationalization experience within firms does not alleviate the occurrence of foreign divestment for multinational enterprises (*β*_*Exp*_ = 0.004, P < 0.1). This phenomenon may be elucidated from the perspective that the accumulation of internationalization experience fosters a sense of undue executive overconfidence, thereby detrimentally influencing the survival rate of overseas subsidiaries, as posited by Petersen, Pedersen, & Lyles [78].

Building upon Model 2, Model 3 introduces formal institutional distance and the interaction term between formal institutional distance and cultural friction as moderator variables to examine the moderating effect on cultural friction and foreign divestment. The regression results reveal that the coefficient of the interaction term, cultural friction × formal institutional distance, is -0.085, and it is statistically significant at the 0.1% level. This signifies that formal institutional distance exerts a significant negative influence on the relationship between cultural friction and foreign divestment, indicating that formal institutional distance mitigates the positive impact of cultural friction on foreign divestment. Thus, Hypothesis H2 is empirically supported.

Given that subgroup regression is another commonly employed approach for testing moderating effects, this study categorizes the target sample based on the presence or absence of political connections. Models 4 and 5 are employed to verify the impact of these informal institutional networks on the relationship between cultural friction and foreign divestment. Herein, *PC* = 0 denotes companies without political connections, while *PC* = 1 indicates companies with political connections. The regression outcomes demonstrate that political connections intensify the positive impact of cultural friction on foreign divestment, with a regression coefficient of 0.094 (P<0.05). This demonstrates that political connections increase the risk of divestment of multinational corporations. Importantly, in enterprises without political connections, the influence of cultural friction on foreign divestment lacks statistical significance. This emphasizes the necessity of analyzing the cultural friction-foreign divestment relationship from a political affiliation perspective. The odds ratio (OR) further indicates that for firms with political connections, with every 1% increase in cultural friction, the probability of foreign divestment by multinational enterprises surges by 9.8%. This bolsters the confirmation of the existence of the curse effect of political connections, thereby hypothesis H3 is verified.

### 4.3 Robust test

#### 4.3.1 Alternative moderator variable

The robustness of the model is confirmed by altering the measurement approach for formal institutional distance and political connections. Following the methodology of previous researchers, this study uses the absolute difference method to calculate the institutional distance between the home and the host country [79]. The specific formula is as follows:

$$Inst=\frac{\sum_{K=1}^{N}\left|I_{ki}-I_{kj}\right|}{N}$$ (9)

In which, *Inst* is the formal institutional distance between two countries, *I*_*ki*_ is the score of country i in the kth dimension, *I*_*kj*_ is the score of country j in the kth dimension, and *N* is the number of dimensions in the institutional environment. The regression results demonstrate the existence of the "institutional distance paradox," which states that, when other factors are held constant, the greater the formal institutional distance, the smaller the impact of cultural friction on foreign divestment.

Following the methodology of previous researchers, this paper employs the ratio of government subsidies to corporate revenue as a more accurate measure of government-business relationships. The binary variable used in the preceding text was found to be inadequate in capturing the degree of association within this informal institutional network [80]. To analyze the data, the target sample is divided into two groups–low and high political connections–using the principle of median division. Subsample regressions are then conducted to delve into the specific relationships. The findings reveal that, when other factors are held constant, enterprises with stronger political connections amplify the positive impact of cultural friction on foreign divestment. The regression coefficient for this effect is 0.055, and it is statistically significant at the 10% level (Table 4). This result emphasizes the presence of the "political connections curse effect."

**Table 4:** Change the measurement method of moderator variables.

Model/Variable	Benchmark regression		Regulatory effect test		
	(1)	(2)	(3)	(4)	(5)
	Full sample	Full sample	Full sample	Government subsidy < median	Government subsidy > median
**Explanatory variable**					
***CF***	0.100^***^ (0.000)	0.050^*^ (0.026)	0.330^***^ (0.000)	0.036 (0.318)	0.055^+^ (0.060)
**Moderator variable**					
***Inst***			0.655^***^ (0.000)		
***Inst·CF***			-0.215^***^ (0.000)		
**Control variable**	Control	Control	Control	Control	Control
**Constant**	-2.034^***^ (0.000)	-1.497 (0.179)	-1.939^+^ (0.086)	0.199 (0.906)	-3.197^*^ (0.046)
***LR***	38.91	54.45	90.39	32.29	28.24
***Prob >chi2***	0.000	0.000	0.000	0.000	0.004
***R*** ^***2***^	0.020	0.028	0.046	0.036	0.027

*Note*: This table presents the test results of benchmark regression and moderating effect after a modification to the measurement method of moderator variables. Specifically, formal institutional distance is measured by absolute difference, and political connections are measured by the degree of government subsidy. Consistent with the regression sequence in Table 3, column 2 reports the regression coefficient of cultural friction and foreign divestment, column 3 reports the regression coefficients of the variables after including all the control variables, column 4 reports the regression coefficients after the inclusion of formal institutional distance, and columns 5–6 report the regression results of the variables in groups without and with political connections, respectively. The p-values associated with these coefficients are reported in parentheses.

^+^p<0.1

^*^p<0.05

^**^p<0.01

^***^p<0.001.

#### 4.3.2 Replacing the metrological model

To enhance the robustness and reliability of our regression outcomes, we employ Probit regression in place of Logit regression to reexamine the estimation model [81], as presented in Table 5. Upon a thorough review of the regression results, it becomes clear that all previously stated hypotheses have been convincingly supported. Notably, we find that cultural friction has a significant and positive influence on foreign divestment, providing substantial evidence for the existence of both the "formal institutional distance paradox" and the "political connections curse effect" within the domain of foreign divestment.

**Table 5:** Probit regression result.

Model/Variable	Benchmark regression		Regulatory effect test		
	(1)	(2)	(3)	(4)	(5)
	Full sample	Full sample	Full sample	*PC* = 0	*PC* = 1
**Explanatory variable**					
***CF***	0.059^***^ (0.000)	0.028^*^ (0.029)	0.200^***^ (0.000)	0.009 (0.580)	0.054^*^ (0.020)
**Moderator variable**					
***Inst***			0.163^***^ (0.000)		
***Inst·CF***			-0.051^***^ (0.000)		
**Control variable**	Control	Control	Control	Control	Control
**Constant**	-1.211^***^ (0.000)	-0.928 (0.128)	0.023 (0.803)	-0.597 (0.427)	-1.302 (0.229)
***LR*** ***Prob >chi2*** ***R*** ^***2***^	40.84	55.97	99.03	41.14	19.56
	0.000	0.000	0.000	0.000	0.003
	0.021	0.029	0.051	0.031	0.031

*Note*: This table presents the test results of benchmark regression and moderating effect after a modification to the regression model. The model used in this table is Probit regression, and the regression sequence is consistent with Table 3. Column 2 reports the regression coefficient of cultural friction and foreign divestment, column 3 reports the regression coefficients of the variables after including all the control variables, column 4 reports the regression coefficients after the inclusion of formal institutional distance, and columns 5–6 report the regression results of the variables in groups without and with political connections, respectively. The p-values associated with these coefficients are reported in parentheses.

^+^p<0.1

^*^p<0.05

^**^p<0.01

^***^p<0.001.

### 4.4 Endogeneity testing

#### 4.4.1 Lagged independent variable regression

Following the methodology of Li et al., this study delays cultural friction by one period before regressing. This strategic step is implemented to mitigate potential reverse effects of foreign divestment on cultural friction, effectively addressing concerns related to endogeneity and reverse causality. The application of this one-period lag on *CF* results in a reduction in the number of observations from the original 2385 to 2034, as shown in Table 6. Despite this reduction, the regression outcomes consistently align with the earlier findings. From a friction perspective, the lagged cultural friction still exerts a significant positive impact on foreign divestment by multinational enterprises, with a regression coefficient of 0.120 (P < 0.001). Moreover, from an institutional viewpoint, the formal institutional distance continues to mitigate the positive influence of cultural friction on foreign divestment. Concurrently, the informal institutional relationship of political connections exacerbates the positive impact between them.

**Table 6:** Lagging one-phase test.

Model/Variable	Benchmark regression		Regulatory effect test		
	(1)	(2)	(3)	(4)	(5)
	Full sample	Full sample	Full sample	*PC* = 0	*PC* = 1
**Explanatory variable**					
***CF***_***1***_	0.125^***^ (0.000)	0.120^***^ (0.000)	0.292^***^ (0.000)	0.092^**^ (0.002)	0.180^***^ (0.000)
**Moderator variable**					
***Inst***			0.198^***^ (0.000)		
***Inst·CF***_***1***_			-0.049^**^ (0.012)		
**Control variable**	Control	Control	Control	Control	Control
**Constant**	-1.994^***^ (0.000)	-0.839 (0.465)	-1.229 (0.290)	-0.419 (0.764)	-1.445 (0.487)
***LR***	57.47	63.96	79.88	41.50	25.93
***Prob >chi2***	0.000	0.000	0.000	0.000	0.002
***R*** ^***2***^	0.031	0.035	0.043	0.033	0.045

*Note*: This table presents the test results of benchmark regression and moderating effect following a one-period delay in the independent variable, cultural friction. Specifically, Column 2 reports the regression coefficient of cultural friction and foreign divestment, column 3 reports the regression coefficients of the variables after including all the control variables, column 4 reports the regression coefficients after the inclusion of formal institutional distance, and columns 5–6 report the regression results of the variables in groups without and with political connections, respectively. The p-values associated with these coefficients are reported in parentheses.

^+^p<0.1

^*^p<0.05

^**^p<0.01

^***^p<0.001.

#### 4.4.2 Instrumental variable method

Cultural friction is a product of cultural distance, but as a national-level objective variable, it doesn’t directly impact the foreign divestment of multinational enterprises in the absence of physical interactions. To address potential endogeneity concerns within the model, we have chosen to use cultural distance (*CD*) as an instrumental variable in this study. We’ve followed a method inspired by Bollen, Guilkey, & Mroz for assessing endogeneity in a binary choice model. Our research has undertaken preliminary instrumental variable tests and weak instrument tests [82] to identify endogenous variables within the model and assess the appropriateness of instrumental variable selection, as detailed in Table 7. First, the results of the second-stage regression in the initial instrumental variable test reveal a highly significant p-value of 0.000, signifying at the 0.1% significance level that *CF* is indeed an endogenous explanatory variable. Moreover, when we consider endogeneity issues, the positive impact of cultural friction on foreign divestment is substantially underestimated. The original regression coefficient for *CF* was 0.050 (P < 0.05), whereas accounting for endogeneity concerns led to a revised coefficient of 0.448 (P < 0.001). Lastly, the first-stage regression results highlight the robustness of the instrumental variable *CD*, with a statistical value of 0.528 (P < 0.001). Second, based on the results of the weak instrument test, both the AR statistic and the Wald statistic are significant at the 0.1% level, indicating that the instrumental variables chosen in this study are not weak and satisfy the criteria for instrumental variable selection. These findings reinforce the validity of our results and confirm the consistency of the regression outcomes with our earlier discussion.

**Table 7:** Test result of instrumental variable method.

First stage regression results I			First stage regression results II		Second stage regression result		
Independent variable	Statistical value		Independent variable	Statistical value	Independent variable		Statistical value
***CD***	0.528^***^ (0.000)		***CD***	-0.417 (0.112)	***CF***		0.448^***^ (0.000)
***CD·Inst***	-0.021 (0.135)		***CD·Inst***	-0.544^***^ (0.000)	***CF·Inst***		-0.056^**^ (0.001)
***Size***	0.116^***^ (0.000)		***Size***	0.413^**^ (0.002)	***Size***		0.023 (0.377)
***Age***	0.027 (0.888)		***Age***	-0.512 (0.510)	***Age***		-0.540 (0.000)
***Exp***	0.068^***^ (0.000)		***Exp***	0.254^***^ (0.000)	***Exp***		-0.012^**^ (0.002)
***Own***	-0.076 (0.552)		***Own***	0.299 (0.559)	***Own***		0.079 (0.425)
***Ind***	0.009 (0.454)		***Ind***	0.072 (0.141)	***Ind***		-0.002 (0.795)
F = 372.49 R^2^ = 0.536			F = 336.34 R^2^ = 0.510		Chi^2^ = 24.06 ^***^P = 0.000		
***AR***		32.40^***^(0.0000)		***Wald***		29.72^***^(0.0000)	

*Note*: This table presents the test results of preliminary instrumental variable tests and weak instrument tests. The instrumental variable selected in this study is cultural distance, and columns 1, 3 and 5 all represent the name of the variables. Notably, columns 2 and 4 report the regression results of the first stage, where the dependent variable of result I is cultural friction, the dependent variable of result II is the cross term of cultural friction and formal institutional distance. Column 6 reports the second-stage regression results, with foreign divestment as the dependent variable. The p-values associated with these coefficients are reported in parentheses.

^+^p<0.1

^*^p<0.05

^**^p<0.01

^***^p<0.001.

## 5 Further analysis

Foreign divestment, being a strategic decision made by a parent company based on the unique traits of its overseas subsidiaries, exhibits strong individual effects. However, existing research has primarily concentrated on how the overall level of cultural friction faced by the parent company impacts its divestment activities, which falls short in addressing the impact of cultural friction on the survival rate of individual foreign subsidiaries. To address this gap, in the subsequent analytical phase, this study shifts its emphasis to the subsidiary level. Here, we investigate the influence of cultural friction on the survival rate of each overseas subsidiary. A lower survival rate of foreign subsidiaries corresponds to a higher risk of divestment, presenting a valuable augmentation to the preceding research.

### 5.1 Data sources

The upcoming study exclusively utilizes cross-sectional data for specific firms due to the limited annual variation in cultural friction at the individual subsidiary level. In this scenario, each subsidiary represents a distinct data point. We obtained subsidiary records for publicly listed firms from the CSMAR database and selected a sample of 893 overseas subsidiaries established by 422 Chinese enterprises between 2000 and 2021.The remaining data were obtained through a similar channel as previously mentioned. Details of the data can be found in S2 Dataset.

### 5.2 Model selection

Since the Logit regression used above cannot control the individual effects of the samples, we introduce the Cox survival proportional hazards model to explore the relationship between cultural friction and foreign divestment. As a semi-parametric model, Cox proportional hazards model is commonly employed to assess the probability of survival for foreign subsidiaries [83]. In this study, the divestment risk rate of overseas subsidiaries constitutes the explained variable. To measure this indicator, two types of data are essential: survival status and survival time. In our study, if an overseas subsidiary withdrew from the foreign market during the observation period, we considered it a business failure and assigned it a value of 1. Conversely, if the subsidiary remained in the foreign market, it was assigned a value of 0. The survival time represents the duration from the subsidiary’s entry into the host country market to its exit. For subsidiaries that did not withdraw during the observation period, the survival time is calculated as the difference between 2021 and the establishment year. The model posits that various survival risks faced by enterprises may lead to their exit from the market, consequently triggering divestment actions by the parent company. The fundamental formulation is expressed as Eq (10):

$$h(t,X)=h_{0}(t)\exp(\sum_{i=1}^{m}\beta_{i}x_{i})$$ (10)

In which, *h* (*t*, *X*) is the survival risk function of a corporation, while h_0_(t) is the baseline risk solely associated with time *t*. The coefficient *β* is the marginal effects of various variables on the subsidiary divestment risk, which can be computed through maximizing the partial likelihood function. The covariate vector *X* = (*x*_1_,*x*_2_…*x*_m_) is an amalgamation of diverse risk factors influencing corporate survival. The term exp(*β*_i_*x*_i_) is the actual risks confronted by the corporation. As the explanatory variable x_i_ increases by one unit, the hazard ratio (HR) transforms by a factor of exp(*β*_i_). Specifically, when HR>1, it indicates that the instantaneous risk of divestment for overseas subsidiaries escalates with the augmentation of the independent variables. For HR = 1, the instantaneous divestment risk of overseas subsidiaries remains unaffected by the independent variables. When HR<1, it signifies that the divestment risk ratio diminishes with the amplification of the independent variables.

It’s important to emphasize the requirement of meeting the proportional hazards assumption when applying the Cox model. However, in the context of global commerce, satisfying this assumption proves to be quite challenging. In such scenarios, the introduction of a time-dependent covariate Cox regression model, also known as a non-proportional hazards model, becomes necessary. Through the assessment of the proportional hazads (PH) assumption, it is evident that variables such as cultural friction, internationalization experience, industry property and firm age, do not adhere to the PH assumption (P<0.05). Hence, the adoption of a Cox model that incorporates time-dependent variables is imperative. The fundamental formulation is presented in Eq (11), where the covariate values change over time while the other aspects remain consistent with the traditional fixed Cox model.


$$h(t,X)=h_{0}(t)\exp(\sum_{i=1}^{m}\beta_{i}x_{i}(t))$$ (11)

### 5.3 Data analysis

#### 5.3.1 Descriptive statistics

Table 8 presents the geographical distribution of the overseas subsidiary sample. Most of the target subsidiaries are situated in Europe and Asia, accounting for a combined total of 79.6%. Regarding host countries, these subsidiaries are distributed across 49 different nations, with a significant concentration in countries like the United States, Singapore, and Germany. Notably, there are 237 overseas subsidiaries located in the United States, constituting approximately 26.5%. (Table 8 provides a partial listing of host countries with a count not less than 10).

**Table 8:** Distribution of host countries of overseas subsidiaries.

Region	Number	Ratio	Region	Number	Ratio
**Asia**	267	29.9%	**Europe**	216	24.2%
Philippines	10	1.12%	Luxembourg	11	1.23%
Indonesia	18	2.02%	Switzerland	11	1.23%
Thailand	19	2.13%	Russia	13	1.46%
India	26	2.91%	Spain	14	1.57%
Malaysia	26	2.91%	France	15	1.68%
Viet Nam	28	3.14%	Britain	27	3.02%
South Korea	34	3.81%	Italy	34	3.81%
Japan	46	5.15%	Netherlands	38	4.26%
**South America**	11	1.23%	**Oceania**	40	4.48%
Brazil	11	1.23%	Australia	40	4.48%
**North America**	270	30.2%			
Canada	33	3.70%			
USA	237	26.5%			

*Note*: This table presents summarized information on the distribution of the target subsidiaries. The region name displays in columns 1 and 4, the number of subsidiaries displays in columns 2 and 5, and the ratio of the number of subsidiaries in each region to the overall sample displays in columns 3 and 6.

To mitigate the impact of outliers on the analysis outcomes, this study utilized Stata to winsorize variables before conducting a descriptive analysis. The results are presented in Table 9, revealing important insights. Specifically, the maximum value for cultural friction is 11.419, while the minimum value is 0, highlighting substantial disparities in cultural friction among the sampled companies. Notably, the level of internationalization varies significantly, with a high standard deviation of 46.128. The data of variable correlation analysis and collinearity test are shown in S2 Table.

**Table 9:** Descriptive statistics of variables.

Variable name	Variable symbol	Sample size	Average	Standard deviation	Minimum	Maximum
Foreign divestment	*FD*	893	0.353	0.478	0	1
Cultural friction	*CF*	893	1.212	1.791	0	11.419
Firm size	*Size*	886	22.173	1.454	16.587	26.816
Firm age	*Age*	893	28.290	2.205	24	34
International experience	*Exp*	893	27.896	46.128	0	460
Firm Ownership	*Own*	893	0.159	0.366	0	1
Industry property	*Ind*	893	4.496	3.377	1	18
Formal institutional distance	*Inst*	893	1.201	0.432	0.585	1.843
Political connections	*PC*	893	0.353	0.478	0	1

*Note*: This table presents descriptive statistics for all variables, with columns 1 to 7 representing variable name, variable symbol, sample size, average value, standard deviation, and Max-min value, respectively.

#### 5.3.2 Non-parametric analysis

Table 10 offers statistics on the survival times of the sample companies, highlighting that the majority, approximately 12.43%, consists of foreign subsidiaries with a survival duration of five years. The next highest proportion corresponds to companies with a four-year survival time, accounting for around 8.85%. As for the remaining survival times, the percentages range from 0.78% to 8.62%. In Table 11, you’ll find the annual counts of survival and exits for the overseas subsidiaries over the observation years. The results reveal that the most significant decline in survival rate occurs during the fifth year, with a decline ratio of 0.075. The second-highest decline takes place in the second year, with a decline ratio of 0.066. The decline ratios for the remaining years range from 0 to 0.060.

**Table 10:** Statistics on the life span of overseas subsidiaries.

Survival time	Number of overseas subsidiaries	Proportion	Survival time	Number of overseas subsidiaries	Proportion
1	78	8.73%	12	34	3.81%
2	67	7.50%	13	25	2.80%
3	77	8.62%	14	28	3.14%
4	79	8.85%	15	22	2.46%
5	111	12.43%	16	13	1.46%
6	70	7.84%	17	7	0.78%
7	50	5.60%	18	8	0.90%
8	50	5.60%	19	6	0.67%
9	47	5.26%	20	5	0.56%
10	44	4.93%	21	12	1.34%
11	53	5.94%	22	7	0.78%

*Note*: This table presents the sample companies’ survival times. Columns 1 and 4 report the survival time of the target subsidiaries, columns 2 and 5 report the number of subsidiaries corresponding to each survival time, and columns 3 and 6 report the proportion of the number of subsidiaries corresponding to each survival time to the total sample.

**Table 11:** K-M estimator of overseas subsidiaries.

Survival time (year)	1	2	3	4	5	6	7	8	9	10	11
**Number of subsidiaries**	893	815	748	671	592	481	411	361	311	264	220
**Exit number**	19	43	53	47	55	23	21	16	9	13	4
**K-M estimated value**	0.979	0.927	0.861	0.801	0.726	0.691	0.656	0.627	0.609	0.579	0.568
**Survival time (year)**	12	13	14	15	16	17	18	19	20	21	22
**Number of subsidiaries**	167	133	108	80	58	45	38	30	24	19	7
**Exit number**	1	0	4	2	1	3	0	0	0	1	0
**K-M estimated value**	0.565	0.565	0.544	0.530	0.521	0.486	0.486	0.486	0.486	0.461	0.461

*Note*: This table presents the K-M estimator of overseas subsidiaries. Lines 1 and 5 report the survival time of overseas subsidiaries, lines 2 and 6 report the number of overseas subsidiaries corresponding to each survival time, lines 3 and 7 report the number of exiting subsidiaries corresponding to each survival time, and lines 4 and 8 report the K-M estimates.

**Fig 2:**
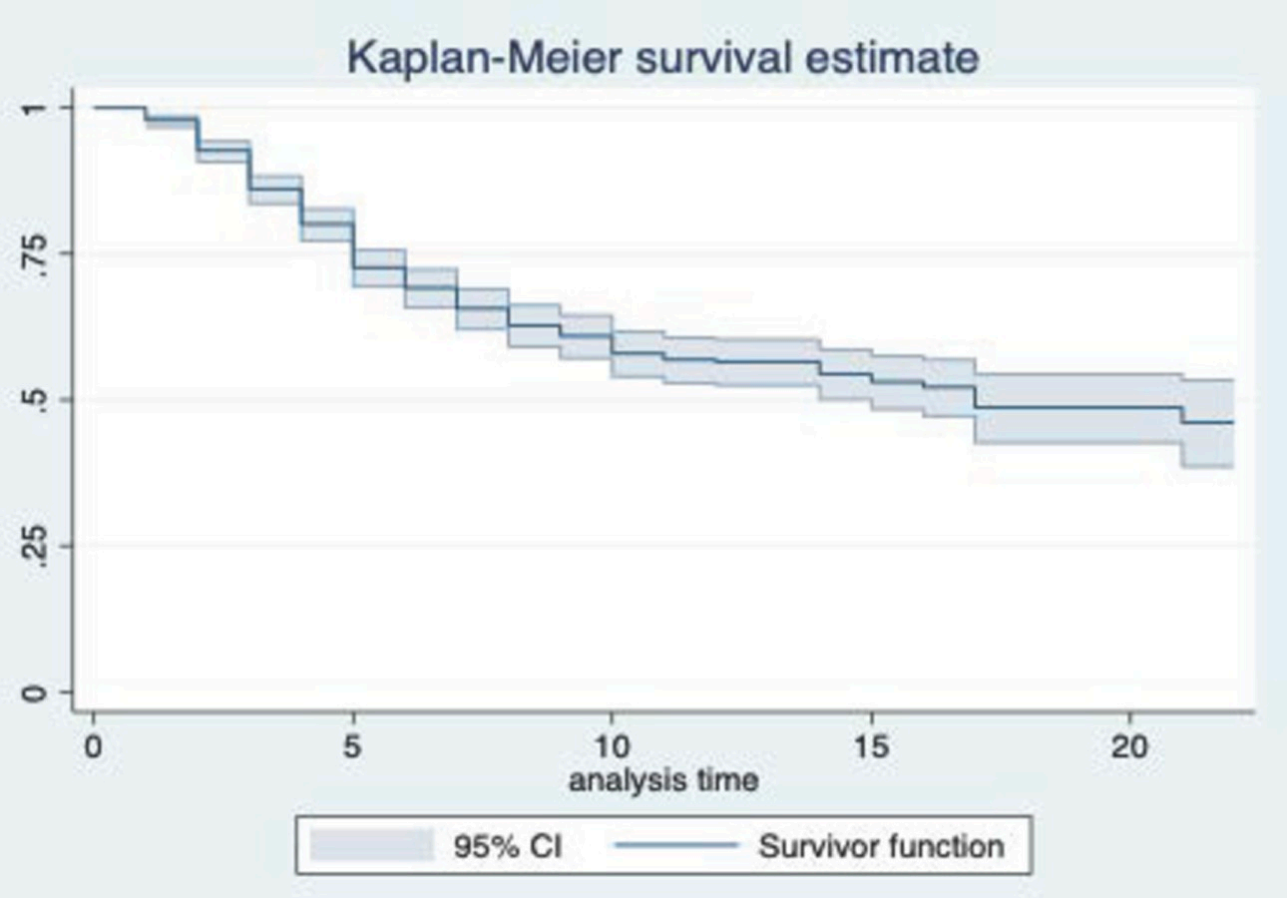
Kaplan-Meier survival function diagram.

**Fig 3:**
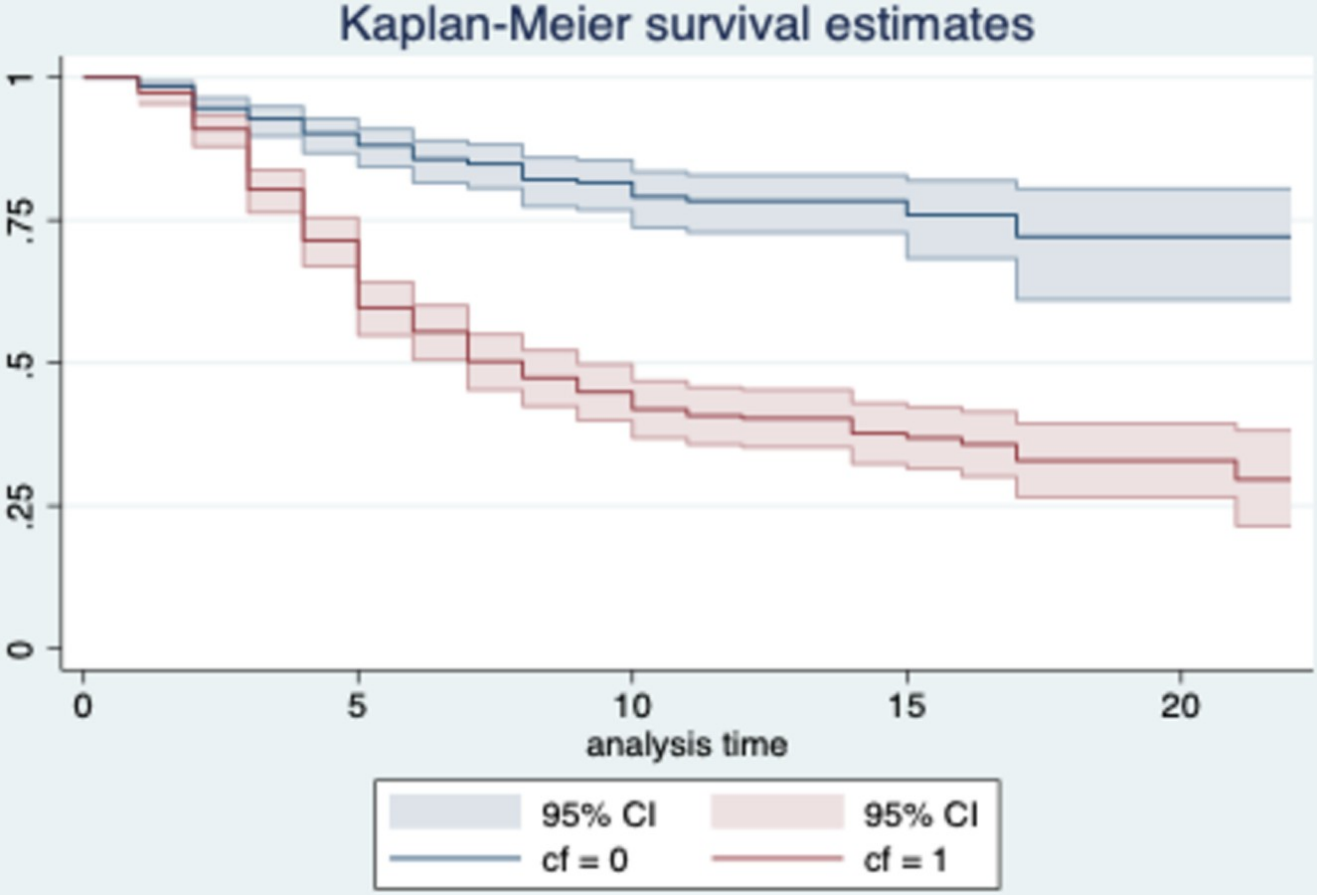
Kaplan-Meier group survival function diagram.

Building upon the approach delineated by Lim, this study further divided the target sample into two subsamples based on the median values of cultural friction, aiming to investigate potential differences in survival functions between these groups [84]. The results of the Log-rank test reveal statistically significant differences in the survival distributions among the cultural friction groups (χ^2^ = 102.51, P<0.000). Furthermore, by examining the Kaplan-Meier survival curves (Figs 2 and 3), it becomes evident that the curve for the subsample with cultural friction values below the median (CF = 0) is higher than the curve for the subsample with cultural friction values above the median (CF = 1). This finding implies that the survival rate of foreign subsidiaries decreases as cultural friction increases.

#### 5.3.3 Cox regression analysis

In this study, we employed a time-dependent Cox model for our analysis. The empirical results of this analysis are presented in Table 12. As discussed in our variable inclusion process, Model 1 exclusively incorporates the independent variable, cultural friction. The results show a positive coefficient for cultural friction (*β*_*CF*_ = 0.223, P = 0.000) with a hazard ratio of 1.250 (e^0.223^). This implies that for each incremental unit increase in cultural friction, the instantaneous death risk rate of overseas subsidiaries increases by 1.250. In essence, an elevation in cultural friction negatively impacts the survival rate of overseas subsidiaries, consequently promoting the occurrence of parent company divestment. As a result, Hypothesis H1 is confirmed.

**Table 12:** Benchmark regression and moderating effect test results.

Model/Variable		Benchmark regression		Regulatory effect test		
		(1)	(2)	(3)	(4)	(5)
		Full sample	Full sample	Full sample	PC = 0	PC = 1
		Coefficient	Coefficient	Coefficient	Coefficient	Coefficient
***main***	**Explanatory variable**					
	***CF***	0.223^***^ (0.000)	0.104^*^ (0.031)	0.393^***^ (0.000)	0.202^***^ (0.000)	0.315^***^ (0.000)
	**Moderator variable**					
	***Inst***			-0.175 (0.478)		
	***Inst·CF***			-0.104^*^ (0.022)		
	**Control variable**					
	***Size***		0.141^**^ (0.002)	0.140^**^ (0.003)	0.196^**^ (0.001)	-0.036 (0.669)
	***Age***		0.019 (0.495)	0.019 (0.519)	-0.024 (0.526)	-0.106 (0.392)
	***Exp***		0.005 (0.219)	0.001 (0.765)	0.002 (0.602)	-0.018^**^ (0.004)
	***Own***		-1.799^**^ (0.001)	-1.505^**^ (0.004)	-1.494^**^ (0.009)	-0.165 (0.665)
	***Ind***		-0.000 (0.987)	-0.001 (0.963)	0.016 (0.476)	0.005 (0.882)
***tvc***	***CF***		0.086^**^ (0.003)			
	***Exp***		-0.007^**^ (0.007)	-0.003 (0.233)	-0.003 (0.258)	
	***Own***		0.870^**^ (0.004)	0.736^*^ (0.014)	0.713^*^ (0.027)	
	***Age***					0.127 (0.108)
***Sample number***		893	886	886	577	309
***Log-likelihood***		-1924.7614	-1890.3079	-1889.0545	-1139.0425	-541.7983
***LR***		119.43	158.26	160.77	103.11	68.99

*Note*: This table presents the test results of benchmark regression and moderating effect in Cox regression model. The Cox regression coefficients for each variable are reported in the main section, and the regression coefficients for the variables that do not satisfy the PH hypothesis are reported in the tvc section. Specifically, the coefficient in column 3 is obtained from the Cox regression of cultural friction and foreign divestment. Column 4 reports the regression coefficients of the variables after including all the control variables, column 5 reports the regression coefficients after the inclusion of formal institutional distance, and columns 6–7 report the regression results of the variables in groups without and with political connections, respectively. The p-values associated with these coefficients are reported in parentheses.

^+^p<0.1

^*^p<0.05

^**^p<0.01

^***^p<0.001.

Model 2 extends upon Model 1 by incorporating all control variables. Notably, it’s essential to recognize that three variables, CF, Exp and Own, do not adhere to the PH assumption (with time-dependent p-values all below 0.05), necessitating the consideration of time-dependent effects. Taking CF as an example, the coefficient for CF is 0.104. The formula for computing the hazard ratio is HR_*Exp*_ = exp(0.104+0.086·ln(t)), where t represents time. For instance, when t = 10, HR_*Exp*_ = exp (0.104+0.086·ln (10)) = 1.352. This implies that when the survival time of overseas subsidiaries is 10 years, for each unit increase in CF, the divestment risk rate of overseas subsidiaries increases by 35.2%. This observation aligns with the analysis conducted at the parent company level.

Model 3 builds upon Model 2 by introducing the first moderator variable, formal institutional distance. The data outcomes reveal that the coefficient for the interaction term between cultural friction and formal institutional distance is -0.104 (P = 0.022). This finding indicates that formal institutional distance negatively moderates the relationship between cultural friction and the divestment risk of overseas subsidiaries. In other words, formal institutional distance weakens the positive connection between cultural friction and the divestment risk of foreign subsidiaries for multinational corporations, thereby confirming Hypothesis H2.

Another widely used technique for examining moderating effects is grouping regression. In our study, we grouped the target firms based on the presence or absence of political connections. Models 4 and 5 are employed to explore the influence of cultural friction on the survival relationship of overseas subsidiaries for firms without political connections and those with political connections, respectively. Our findings show that the sample of businesses with political connections had a higher instantaneous death risk rate (1.370) compared to businesses without political connections (risk ratio: 1.224). Revealing that as cultural friction increases, the divestment risk rate of overseas subsidiaries with political connections is higher. Hypothesis H3 is confirmed.

## 6 Conclusion and discussion

### 6.1 Conclusion

Drawing upon data from the foreign investments of Chinese listed companies, this study conducts a quantitative investigation into the relationships between cultural friction, formal institutional distance, political connections, and the divestment behavior of multinational enterprises (MNEs). To explore the distinct impacts of these variables at the parent firm and subsidiary levels, the study employs both the panel random effects Logit regression model and Cox survival analysis. The findings reveal the following key insights: Firstly, cultural friction has a positive influence on the rate of foreign divestment, whether at the parent firm or overseas subsidiary level. In simpler terms, higher cultural friction leads to lower survival rates of overseas subsidiaries and an increased rate of foreign divestment for multinational enterprises. Secondly, from an institutional perspective, foreign divestment by multinational enterprises is shaped by both formal and informal institutional factors. There exist the "formal institutional distance paradox" and the "political connections curse effect." Formal institutional distance moderates the positive impact of cultural friction on foreign divestment, while political connections, as informal institutional factors, bolster the confidence of multinational enterprises in expanding recklessly, thereby intensifying the positive influence of cultural friction on foreign divestment. These patterns are consistent at both the parent company and subsidiary levels. Lastly, robustness tests underscore that while cultural distance can reflect cultural distinctions between two countries, it falls short in capturing the varied cultural resistance faced by different firms within the same host country. Thus, cultural friction emerges as a more effective metric. The study also replicates its main models by altering measures of moderator variables and econometric specifications, with results consistently aligned with the original hypotheses.

### 6.2 Theoretical contributions

The research results contribute to existing theories in the following ways: Firstly, by introducing the concept of cultural friction into the study of foreign divestment, this research validates the positive influence of cultural friction on foreign divestment, building upon the theory of LOF. It is well-established in academia that differences in political, economic, and cultural aspects among countries impact multinational corporations’ foreign investment behavior [85,86]. While some scholars have explored this topic from the angles of political and economic friction, confirming their effects on foreign divestment, the softer influence of cultural friction has often been overlooked. In this context, our study places cultural friction at the center of attention. We utilize the perspectives of information asymmetry [87] and cognitive legitimacy deficit [43] to dissect the internal mechanisms through which cultural friction affects the foreign divestment of multinational enterprises. We investigate both the parent company and subsidiary levels, employing empirical data to validate our hypothesis that cultural friction heightens the likelihood of foreign divestment. This study makes a substantial theoretical contribution by broadening our comprehension of cultural friction in global markets and elucidating how individual cultural resistance shapes the foreign divestment process. Secondly, from an institutional perspective, this study delves into the boundary factors that influence the relationship between cultural friction and foreign divestment. First, it utilizes the concept of formal institutional distance to represent the formal institutional pressures faced by multinational enterprises. Our analysis supports the validity of the "formal institutional distance paradox" in the context of foreign divestment, aligning with the logic of both traditional and new institutional theories [88]. This fresh perspective offers new insights into revealing the latent value of formal institutional distance. Second, the study introduces political connections as informal institutional relationships autonomously developed by enterprises. It unveils the rationale behind the "political connections curse effect" from the perspectives of "over-investment" and "political burdens." The research demonstrates that the political connections of multinational enterprises may amplify the positive role of cultural friction in foreign divestment, exacerbating the disadvantages faced by foreign businesses [58,62]. These findings provide novel theoretical inspiration for enhancing the management of business-political connections.

### 6.3 Management implication

This paper offers valuable managerial insights in several key areas: First, Control Cultural Friction: Companies can take proactive steps to reduce cultural friction by understanding the unique cultural systems of their investment destinations and planning their international development. Cultural friction, as a significant driver of foreign divestment, can exacerbate foreigner disadvantage and create cross-border barriers. For example, the Myitsone Dam project faced backlash due to its location conflicting with the cultural beliefs of the Kachin people. Managers are encouraged to focus on the specific cultural aspects of their investment destinations, consider cultural differences between nations, and adapt their international investment strategies accordingly. This preemptive control of cultural friction can mitigate the negative impact of high-intensity cultural resistance on overseas expansion and reduce involuntary foreign divestment. Second, Leveraging Formal Institutional Void: Acknowledge the distinctions among formal institutions and explore the special role of formal institutions in easing the relationship between cultural resistance and foreign divestment. In some case, formal institutional differences can act as a protective mechanism for foreign divestment. Building upon this, the study posits that multinational enterprises should proactively exploit institutional voids and capitalize on external objective institutional advantages to alleviate the adverse impact of cultural resistance on overseas operations. It’s worth noting that we should be cautious about any institutional hazards in less developed places even as we take advantage of the institutional gap. Third, Caution in Building Informal Institutional Networks: While strong political-business relationships are common in China’s business culture, this study reveals that informal links between government and business can worsen the outsider disadvantage faced by multinationals. Therefore, managers of multinational enterprises should carefully understand, obtain, and utilize the policy convenience and financial support provided by informal political connections. They should also clarify their development strategies and goals to avoid blind expansion and enhance decision-making rationality.

### 6.4 Research limitations and prospects

A few limitations need to be acknowledged in the interests of advancing future research. Firstly, this paper only focuses on the impact of cultural friction on foreign divestment, but foreign divestment will also be affected by political, economic and other factors when conducting business overseas. In the future, we can explore these factors’ respective mechanisms and investigate their combined influence. For example, future research could examine the collective impact of political, economic, and cultural friction on businesses’ foreign divestment, which holds great potential [89]. Secondly, concerning the measurement of friction, while our approach combines national distance and firm interaction to address single-country related issues, it primarily emphasizes the combination of national cultural variations and firm-level internationalization. This approach falls short of adequately reflecting the internal cultural institutional arrangements within firms. In the future, researchers can place greater emphasis on designing organizational internal cultural systems and identifying "lubricants" that can mitigate friction. This will expand and refine the research paradigm of friction theory. Thirdly, this analysis neglects the effects of foreign divestment on the parent business or other subsidiaries as it predominantly focuses on the antecedent variables driving foreign divestment. Future studies could consider subsidiary divestment as a moderator variable and investigate its reciprocal impact on the parent company and other interest economies. Additionally, when considering the local and international markets as a whole, foreign divestment by multinational enterprises has broader implications for the local enterprise landscape, which warrants further exploration [90]. Lastly, our findings may lack some generalizability. This study exclusively examines the influence of cultural friction on the foreign divestment of Chinese multinational enterprises. However, the phenomenon of foreign companies withdrawing from the Chinese market is not unique to Chinese firms. In the future, expanding the sample to include representative developed and emerging countries could offer new insights into understanding the relationship between cultural friction and foreign divestment.

## Supporting information

S1 Table Correlation analysis and collinearity test at parent company level. (DOCX)

S2 Table Correlation analysis and collinearity test at subsidiary level. (DOCX)

S1 Dataset Empirical data at parent company level. (XLSX)

S2 Dataset Empirical data at subsidiary level. (XLSX)
