# Supplementary material for: The influence of cultural friction on foreign divestment of multinational enterprises——the moderating role of formal institutional distance and political connections
Source: PLoS One. 2024 Feb 9;19(2):e0295443. doi: 10.1371/journal.pone.0295443 (PMC10857733; doi:10.1371/journal.pone.0295443)
Supplement: S1 Table — (DOCX) [file pone.0295443.s001.docx]

**Supporting information**

### Correlation analysis and collinearity test (analysis of empirical results of subsidiaries)

To mitigate the risk of multicollinearity, this study conducted Pearson correlation coefficient tests on the key explanatory variables (Refer to S4 Table). The results indicate that the maximum correlation coefficient is 0.523, significantly lower than the threshold of 0.8, suggesting the absence of severe multicollinearity issues among the variables. To ensure the robustness of the Pearson correlation coefficient test results, the study also conducted variance inflation factor (VIF) tests on the relevant variables. The outcomes reveal that all variable VIF values are significantly below 10, and the tolerances (1/VIF) are substantially above 0.1. These results collectively indicate the absence of severe multicollinearity problems among the variables.

S4 Table. Correlation coefficient matrix

| **Variable** | ***FD*** | ***CF*** | ***Age*** | ***Exp*** | ***Own*** | ***Size*** | ***Ind*** | ***Inst*** | ***PC*** |
| --- | --- | --- | --- | --- | --- | --- | --- | --- | --- |
| ***FD*** | 1.000 |  |  |  |  |  |  |  |  |
| ***CF*** | 0.523^***^ | 1.000 |  |  |  |  |  |  |  |
| ***Age*** | -0.075^**^ | -0.181^***^ | 1.000 |  |  |  |  |  |  |
| ***Exp*** | 0.051 | 0.381^***^ | -0.243^***^ | 1.000 |  |  |  |  |  |
| ***Own*** | -0.020 | 0.079^**^ | -0.070^**^ | 0.330^***^ | 1.000 |  |  |  |  |
| ***Size*** | 0.089^***^ | 0.235^***^ | -0.190^***^ | 0.408^***^ | 0.299^***^ | 1.000 |  |  |  |
| ***Ind*** | -0.059^*^ | -0.073^**^ | -0.070^**^ | 0.042 | 0.067^**^ | -0.017 | 1.000 |  |  |
| ***Inst*** | 0.004 | -0.006 | 0.020 | 0.016 | 0.020 | -0.041 | 0.016 | 1.000 |  |
| ***PC*** | -0.005 | -0.011 | -0.032 | 0.090^***^ | -0.071^**^ | 0.050 | 0.015 | 0.039 | 1.000 |

Note: *^+^*p<0.1, *^*^* p<0.05, *^**^*p<0.01, *^***^*p<0.001; N=893
